# ULK1-mediated phosphorylation of ATG14 promotes autophagy and is impaired in Huntington’s disease models

**DOI:** 10.1186/s13024-016-0141-0

**Published:** 2016-12-09

**Authors:** Mitchell S. Wold, Junghyun Lim, Véronik Lachance, Zhiqiang Deng, Zhenyu Yue

**Affiliations:** 1 Department of Neurology, The Friedman Brain Institute, Icahn School of Medicine at Mount Sinai, New York, NY 10029 USA; 2 Present Address: Genentech, Inc, 1 DNA Way, South San Francisco, CA 94080 USA; 3 Key Laboratory of Combinatorial Biosynthesis and Drug Discovery, Ministry of Education, School of Pharmaceutical Sciences, and Medical Research Institute, Wuhan University, Wuhan, 430071 China; 4 Leon and Norma Hess Center for Science and Medicine, 9-106, 1470 Madison Ave, New York, NY 10029 USA

**Keywords:** ATG14, Vps34, ULK1, Autophagy, Huntington’s

## Abstract

**Background:**

Autophagy is a bulk degradation pathway for long-lived proteins, protein aggregates, and damaged organelles. ULK1 protein kinase and Vps34 lipid kinase are two key autophagy regulators that are critical for autophagosome biogenesis. However, it isn’t fully understood how ULK1 regulates Vps34, especially in the context of disease. Polyglutamine expansion in huntingtin (Htt) causes aberrant accumulation of the aggregated protein and disrupts various cellular pathways including autophagy, a lysosomal degradation pathway, underlying the pathogenesis of Huntington’s disease (HD). Although autophagic clearance of Htt aggregates is under investigation as therapeutic strategy for HD, the precise mechanism of autophagy impairment remains poorly understood. Moreover, in-vivo assays of autophagy have been particularly challenging due to lack of reliable and robust molecular biomarkers.

**Method:**

We generated anti-phosphorylated ATG14 antibody to determine ATG14-mediated autophagy regulation; we employed Huntington’s disease (HD) genetic cell models and animal models as well as autophagy reporter animal model to understand autophagy signaling and regulation in vivo. We applied biochemical analysis and molecular biology approaches to dissect the alteration of autophagy kinase activity and regulation.

**Results:**

Here, we demonstrate that ULK1 phosphorylates ATG14 at serine 29 in an mTOR-dependent manner. This phosphorylation critically regulates ATG14-Vps34 lipid kinase activity to control autophagy level. We also show that ATG14-associated Vps34 activity and ULK1-mediated phosphorylation of ATG14 and Beclin 1 are compromised in the Q175 mouse model of Huntington’s disease. Finally, we show that ATG14 phosphorylation is decreased during general proteotoxic stress caused by proteasomal inhibition. This reduction of the specific phosphorylation of ATG14 and Beclin 1 is mediated, in part, by p62-induced sequestration of ULK1 to an insoluble cellular fraction. We show that increased ULK1 levels and phosphor-mimetic mutant ATG14 facilitate the clearance of polyQ mutant in cells.

**Conclusion:**

Our study identifies a new regulatory mechanism for ATG14-Vps34 kinase activity by ULK1, which can be used as valuable molecular markers for in-vivo autophagic activity as well as potential therapeutic target for the clearance of polyglutamine disease protein.

**Electronic supplementary material:**

The online version of this article (doi:10.1186/s13024-016-0141-0) contains supplementary material, which is available to authorized users.

## Background

Macroautophagy (simply referred to as autophagy) is a lysosome degradation pathway involving the synthesis, trafficking, and degradation of autophagic vacuoles, or autophagosomes. Basal autophagy is responsible for the turnover of long-lived proteins, protein aggregates, and damaged organelles, but can also be upregulated to cope with various cellular stressors. In fact, autophagy disregulation has been implicated in many disease states. Several studies of postmortem Huntington’s disease (HD) brains and animal models have indicated altered autophagic activity [1–4]. As a bulk cellular degradation pathway, autophagy has also been extensively studied for its neuroprotective potential through the removal of mutant huntingtin (Htt) [5]. However, the precise pathways involved in autophagy during Huntington’s disease (HD) are still being clarified.

Autophagy is tightly regulated by multiple signaling pathways related to nutrient sensing and cellular stress. ULK1 is a serine/threonine kinase that initiates the autophagy cascade [6]. ULK1 is regulated in part by mTOR and AMPK, which inhibit and activate ULK1, respectively [7]. Immediately downstream of ULK1 is the class III PI 3-kinase, Vps34. Vps34 phosphorylates phosphatidylinositol at the 3′ position to form PI(3)P [8], which serves as a second messenger to facilitate the recruitment of later stage, autophagy-related proteins to the site of autophagosome formation. However, Vps34 activity is not limited to autophagy; it is also involved in endosomal sorting and cytokinesis [9, 10]. Vps34 exists in multiple distinct complexes with Beclin 1 and VPS15 [11], but Vps34 in complex with ATG14 is exclusive to autophagy initiation. Upon autophagy induction, ATG14-Vps34 is recruited to the site of autophagosome biogenesis in an ULK1-dependent manner [12].

HD is a fatal neurodegenerative disease caused by mutations in the Htt gene that code for expanded polyglutamine tracts (polyQ) in the first exon, which causes protein aggregation and neuronal loss throughout the brain, most notably in the striatum and cortex. The precise nature of autophagy alterations in HD is not completely understood. However, increasing autophagy has been shown to facilitate the clearance of mutant Htt aggregates [1, 13]. Therefore, understanding the status of autophagy in the context of HD is crucial for the rational design of autophagy-based therapeutics.

Recent work has aimed at understanding the autophagy pathway in finer detail, during the HD pathogenesis. As part of this, a link between the ULK1 kinase and the autophagy receptor, p62/SQSTM1, has been identified. ULK1 phosphorylates p62 to promote selective autophagy in response to proteotoxic stress [14]. Expression of mutant Htt causes an increase in p62 phosphorylation, which can also facilitate autophagic clearance of polyQ protein. Unexpectedly, loss of p62 actually alleviates toxicity in HD mouse models, pointing to a negative impact of p62 in the disease progression [15]. However, it is not fully understood how ULK1 regulates autophagy, especially in the context of protein aggregate prone neurodegenerative diseases.

Herein, we report a mechanism whereby ATG14-Vps34 activity is regulated by ULK1-mediated phosphorylation of ATG14. This phosphorylation occurs in an mTOR-dependent fashion. In contrast to our previous report of increased ULK1-mediated p62 phosphorylation in animal and cellular HD models [14], we show that ATG14 phosphorylation and ATG14-Vps34 activity is decreased in Q175 mice. Furthermore, decreased ATG14 phosphorylation is observed during general proteotoxic stress, which we propose is caused by recruitment of ULK1 away from ATG14 by p62.

## Methods

### Antibodies and reagents

Vps34 (#4263), Myc (#2276), Actin (#3700), and Beclin 1 pS14 (#84966) were purchased from Cell Signaling Technologies (Danvers, MA). Vps15 (#A302-571A), and NRBF2 (#A301-852A) were purchased from Bethyl Laboratories. ATG14 (MBL; #PD026), polyQ (Merck Millipore; #MAB1574), huntingtin aggregates (Merck Millipore, #MAB5374) Beclin 1 (Santa Cruz Biotechnology; #sc-11427), LC3 (Abcam; #ab48394), Flag-M2 (Sigma-Aldrich; #F1804), p62 (American Research Products; #03-GP62-C), p62 pS409 (in house), ULK1 (Sigma-Aldrich, #A7481), GAPDH (Thermofisher; #2D4A7), and GFP (Roche; #11814460001) were purchased from indicated company. The ATG14 antibody used to detect the purified fragment (a.a. 20–73) was made in house [11]. Anti–phosphorylated ATG14 polyclonal antibody was raised in rabbits using the peptide RDLVD(pS)VDDAEGC as an antigen by Cocalico Biologicals. MG132 (Calbiochem), isopropyl-β-D-thiogalactopyranoside (IPTG; Sigma-Aldrich), lipofectamine 2000 (Invitrogen), EDTA-free protease inhibitor cocktail and phosphatase inhibitor cocktail (Roche Diagnostics), Dynabeads protein G (Invitrogen), NuPAGE Bis-Tris gel system (Invitrogen), QuikChange Lightning Site-Directed Mutagenesis Kit (Agilent Technologies), Hybond-P PVDF membrane (GE Healthcare Life Sciences), BCA Protein Assay Reagent Kit (Pierce), Radioactive [γ-32P]ATP (PerkinElmer), Calf intestinal alkaline phosphatase (New England Biolabs), and TALON metal affinity resin (Clontech) were purchased from the companies listed.

### Plasmids

Myc-ULK1 wildtype and kinase inhibited (K46I) mutant were provided by Dr. Sharon Tooze (London Research Institute). Beclin 1-AsRed, ATG14-GFP, FLAG-ATG14, and myc-Vps34-his-Vps15 plasmids were generated as previously described [11]. ATG14 a.a. 20–73 was provided as a generous gift by Dr. Yanxiang Zhao (Hong Kong Polytechnic University).

### Cell culture

MEF, HEK293T, HCT116, and HeLa cells were maintained in Dulbecco’s modified Eagle’s medium (DMEM, Invitrogen) supplemented with 10% fetal bovine serum (Invitrogen) and 50 μg/mL penicillin and streptomycin (Invitrogen). Wild type and p62 KO MEF cells were provided by Dr. Masaaki Komatsu (Niigata University) [16]. ATG14 KO HCT cells were generously provided by Dr. Richard Youle (National Institute of Neurological Disorders and Stroke) [17] and were maintained in McCoy’s 5A plus media (Invitrogen) supplemented with 10% fetal bovine serum, 1X non-essential amino acids (Invitrogen), and glutamine (4 mM final concentration). PolyQ-mCFP HeLa cells were maintained as previously described [13], which were generous gifts from Dr. Ai Yamamoto (Columbia University). Wildtype and ULK1/2 double KO MEFs were a provided by Dr. Mondira Kundu (St. Jude Children’s Research Hospital). Transient DNA transfection of HEK293T and HeLa cells was performed using Lipofectamine 2000 kit according to the manufacturer’s manual (Invitrogen).

### Immunoblot analysis

All IP’s were performed using 10 mM Tris–HCl pH 7.5, 2 mM EDTA, 100 mM NaCl, 1% NP-40, protease inhibitor cocktail (Roche) and phosphatase inhibitor cocktail (Roche). All other lysates were prepared using 50 mM Tris, pH 7.4, 150 mM NaCl, 1% NP-40, 0.25% sodium deoxycholate, 1 mM EDTA, an protease/phosphatase inhibitors (Roche). Supernatants were collected after centrifugation at 13,000 g for 15 min at 4 °C. Supernatants were subjected to BCA assay and then resolved by SDS-PAGE.

### In vitro lipid kinase assays

Immunoprecipitation was performed with 500 μg protein from post-nuclear supernatant, using antibodies against ATG14 (MBL) overnight at 4 **°**C. Samples were incubated with 30 μl Dynabeads (Life Technologies) for 1– 2 h at 4 **°**C and then washed three times with lysis buffer. Two thirds of the beads were reserved for western blotting, while the remaining third was used for the kinase assay. Beads for the kinase assay were washed once with wash buffer (20 mM HEPES pH 7.4, 1 mM EGTA, 0.4 mM EDTA, 5 mM MgCl_2_, and 0.05 mM DTT) and then resuspended to a final volume of 50 μl with reaction buffer (20 mM HEPES pH 7.4, 1 mM EGTA, 0.4 mM EDTA, 5 mM MgCl_2_, and 0.05 mM DTT, 50 mM cold ATP, 5 mM MnCl_2_, 0.1 mg/ml sonicated phosphatidylinositol). 10 nM wortmannin was included in the indicated controls. The reaction was started with the addition of γ-^32^P-ATP (5 mCi) and the samples were shaken at 37 **°**C for 30 min. Reactions were stopped with 120 μl stop buffer (CHCl_3_/CH_3_OH/HCl at a 10:20:0.2 volume ratio) and then shaken for another 10 min at room temperature. Samples were centrifuged for 5 min at 1000 g. 15 μl of the lower, organic phase was resolved on silica coated TLC plate (Millipore) using CHCl_3_/CH_3_OH/NH_4_OH/H_2_O (86:76:10:14 volume ratio) and visualized with the Typhoon 9400 Variable Imager (GE Healthcare Biosciences).

### In vitro ULK1 kinase assay

Myc-ULK1 WT, myc-ULK1 kinase inhibited, or myc protein was immunopurified from HEK cells using anti-myc antibodies. IPs were washed once with kinase buffer (20 mM HEPES pH 7.4, 12.5 mM beta-glycerophosphate, 25 mM MgCl_2_, 5 mM EGTA, 0.25 mM DTT, 100 μM cold ATP, protease inhibitor cocktail (Roche) and phosphatase inhibitor cocktail (Roche)) and then resuspended to a final volume of 50 μl with reaction buffer including 5 μg of substrate (ATG14 a.a. 20–73 or MBP) and 5 μCi ^32^P-ATP. Reaction was incubated at 37 °C for 30 min. Samples were subject to gel electrophoresis in a 4–12% bis-tris gel. The gel was visualized with the Typhoon 9400 Variable Imager (GE Healthcare Biosciences).

### Protein expression and purification

Expression of His-ATG14 a.a. 20–73 was induced in *E.coli* BL21-CodonPlus (Agilent) cells by growing at room temperature for 16 h with 0.05 mM of IPTG. Bacterial cell lysis and protein purification was done using the TALON metal affinity resin kit in strict accordance with the recommended manufacturers protocol (Clontech).

### Triton X-100 soluble and insoluble assays

Samples were lysed in PBS containing 1% Triton X-100 and phosphatase/protease inhibitors on ice for 30 min. After centrifugation at 15,000 g for 30 min at 4 **°**C, Triton X-100 insoluble fractions were collected. Pellets were washed 4x in lysis buffer. Pellets were further solubilized in PBS containing 1% SDS, 1% Triton X-100, and phosphatase/protease inhibitors at 60 **°**C for 1 h. Triton X-100 insoluble fractions were collected after centrifugation at 15,000 g for 30 min at 4 **°**C. A BCA assay was done on the lysates to ensure equal protein concentrations for western blot. This procedure was described elsewhere [14].

### Fluorescence microscopy

Mice were transcardially perfused with ice cold PBS to remove excess blood, then perfused with cold 4% paraformaldehyde. Brain sections were blocked in PBS containing 3% BSA and 0.1% Triton X-100 for 1 h at room temperature. Sections were then incubated with primary antibodies in blocking buffer overnight at 4 °C. After washing 3 times in PBS sections were incubated with Alexa-conjugated secondary antibody for 1 h at room temperature. Secondary antibody was goat anti-mouse Alexa Fluor 555. After 3 more washes with PBS, sections were mounted with mounting medium (ProLong Gold antifade mountant with DAPI, Invitrogen). Cells were examined under Carl Zeiss upright or invert confocal microscopes (LSM780 system). Images were taken with 63X oil immersion objective lens at room temperature and image acquisition was performed by Zen2012 software.

### Animals

All animal studies were performed in compliance with IACUC (Institutional Animal Care and Use Committee) at Icahn School of Medicine at Mount Sinai. Heterozygous z_Q175 and littermate controls were obtained from the CHDI colony at the Jackson Laboratories. For imaging analysis, GFP-LC3 expressing mice [18] were crossed with z_Q175 mice. All mice used were aged 12–15 months.

### Statistical analysis

Data are presented as mean ± SEM from at least three independent experiments. Statistical analysis was performed with GraphPad Prism v5.0 (Graphpad Software). For data normalized with control group (a value of 1), one sample t-tests against a hypothetical value of 1 were used to compare the difference between the other group and control group. For other non-normalized values, unpaired Student’s t-tests were used. For multifactorial analysis, a two-way ANOVA was used. Bonferroni’s correction was used for multiple comparisons. *P* values <0.05 were considered as statistically significant.

## Results

### ULK1 phosphorylates ATG14 at serine 29

**Fig. 1:**
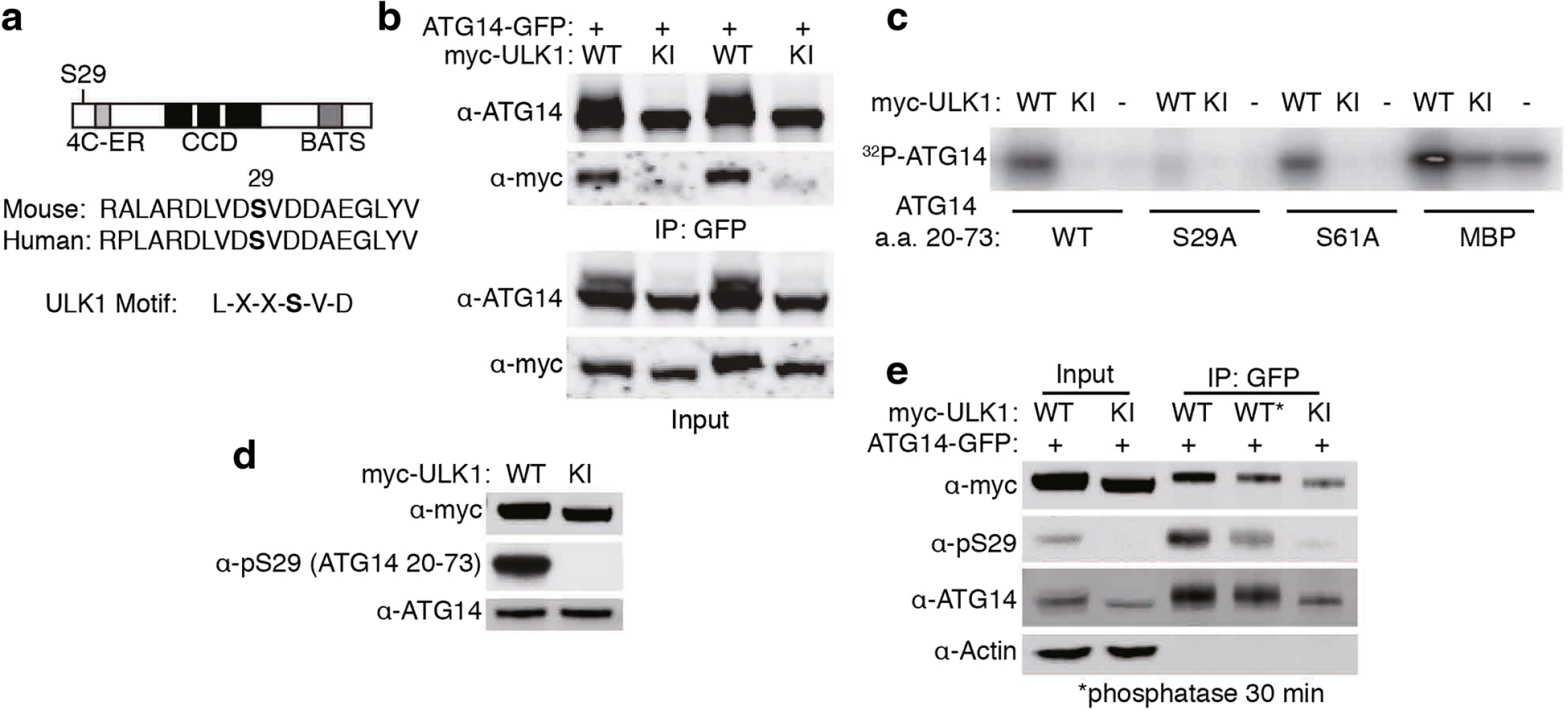
ULK1 phosphorylates ATG14 at serine 29. **a** Representation of ATG14 structure with the location of serine 29. 4C-ER – cysteine repeats needed for ER localization. CCD – coiled-coil domains. BATS – BARKOR autophagosome targeting sequence. **b** Co-immunoprecipitation of ATG14 and ULK1. Myc-ULK1 and ATG14-GFP were overexpressed in HEK cells and pulled down using antibodies against GFP. **c** Autoradiogram from in-vitro kinase assay with ULK1 and ATG14 a.a. 20–73. Myc-ULK1 WT, KI, or empty vector was overexpressed in HEK cells and immunopurified with antibodies against myc. ATG14 a.a. 20–73 WT, S29A, or S61A were purified from e. coli. MBP (myelin basic protein) was used as a positive control. **d** Similar experiment as (**c**), except using an antibody against phosphorylated serine 29. **e** ATG14-GFP and myc-ULK1 WT or KI were co-transfected in HEK cells. Half of the IP was treated with an alkaline phosphatase at 37 °C for 30 min

Previous reports raise the possibility that ULK1 directly regulates ATG14 to influence autophagy [6, 12]. Recent studies have identified a conserved ULK1 phosphorylation consensus sequence [19, 20]. ATG14 contains a similar motif, LXXSVD, at serine 29 (Fig. 1a). Following overexpression of ULK1 and ATG14 in HEK293 cells, we observed that ULK1 wildtype was able to co-immunoprecipitate with ATG14, whereas the interaction of a ﻿mutant ﻿ULK1, kinase-inhibited (KI) was decreased (Fig. 1b). To examine if ULK1 phosphorylates ATG14 at S29, we performed an in vitro kinase assay in which we incubated myc-ULK1 protein and a purified ATG14 fragment (amino acids 20–73) together in the presence of ^32^P labeled ATP. The ATG14 fragment showed incorporation of ^32^P when incubated with ULK1 WT but not with mutant KI (Fig. 1c). In contrast, the S29 alanine mutant fragment showed no incorporation of ^32^P. We also included S61A as a control in the assay, and the result showed no effect of the mutation on ^32^P labeling, suggesting the specificity of S29 phosphorylation by ULK1 in-vitro.

We then developed an antibody against phosphorylated S29 of ATG14, which detected the purified ATG14 fragment in the presence of ULK1 WT but not ULK1 KI mutant (Fig. 1d). In cultured cells, overexpression of ULK1 WT, but not ULK1 KI, increased ATG14 phosphorylation, which is diminished after treatment with an alkaline phosphatase for 30 min (Fig. 1e).

### ATG14 phosphorylation at Ser29 by ULK1 is regulated by the mTOR pathway

**Fig. 2:**
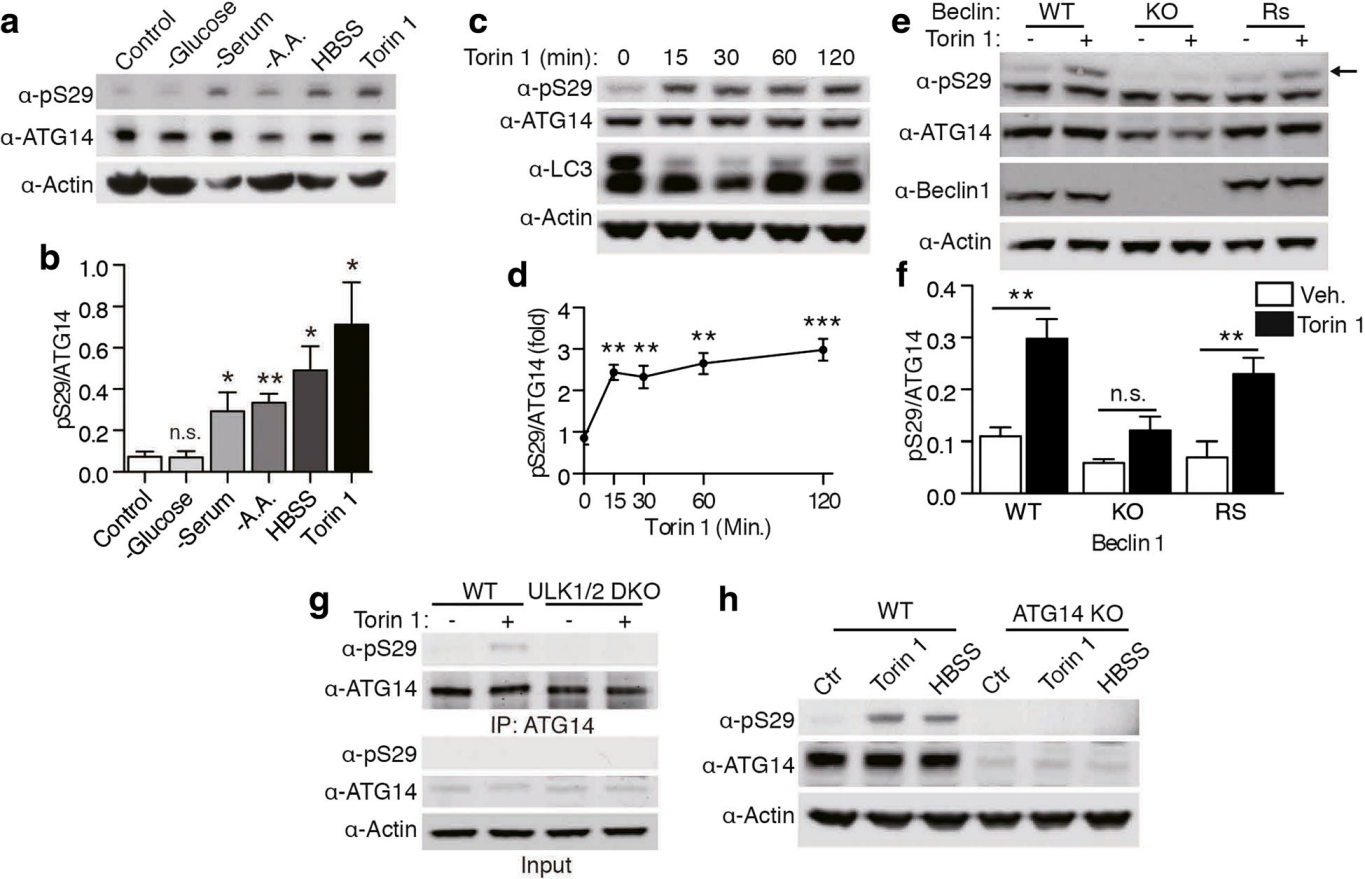
ATG14 is phosphorylated in an mTOR-dependent fashion by ULK1 upon autophagy induction. **a** Phosphorylation of ATG14 in HCT116 cells after glucose, serum, or amino acid withdrawal, medium starvation with HBSS, or Torin 1 treatment for 6 h. **b** ATG14 phosphorylation levels were normalized to total ATG14 levels and compared to the control condition. **p < 0.01 *p < 0.05 n.s. not significant (*n* = 3). Data are represented as mean +/− SEM. **c** ATG14 phosphorylation after 15, 30, 60, and 120 min of Torin 1 treatment in HCT116 cells. **d** ATG14 phosphorylation levels were normalized to total ATG14 levels and compared to the control condition. A one-way ANOVA with Bonferroni’s posttest was performed. F(4, 10) = 12.58, *p* = 0.0006 ***p < 0.001 **p < 0.01 *p < 0.05 (*n* = 3). Data are represented as mean +/− SEM. **e** ATG14 phosphorylation in Beclin WT, KO or Beclin rescued MEF cells treated with Torin 1 or vehicle (DMSO) for 60 min. Arrow indicates pATG14 band. **f** Quantification of ATG14 phosphorylation normalized to total ATG14. **p < 0.01 (*n* = 3). Data are represented as mean +/− SEM. **g** MEF WT and ULK1/2 DKO cells were treated with Torin 1 for 60 min. ATG14 was immunopurified to enhance the phospho-signal. **h** Validation of phospho-ATG14 (S29) antibody specificity. ATG14 phosphorylation signal disappears in ATG14 KO HCT116 cells after medium starvation or Torin 1 treatment for 60 min

We next determined if ATG14 S29 phosphorylation occurs upon autophagy induction. Treatment with Torin 1, a potent mTOR inhibitor [21], caused robust phosphorylation of S29 of ATG14 in HCT116 cells. In addition, amino acid starvation, serum removal, and culture medium starvation all caused an increase in S29 phosphorylation (Fig. 2a, b). Glucose withdrawal, however, did not lead to phosphorylation of ATG14 despite the increase in LC3-II (Additional file 1a), possibly because ULK1/2 -mediated ATG14 phosphorylation is required for amino acid starvation - induced autophagy, but not for glucose withdrawal [22]. Furthermore, a time course analysis showed that ATG14 is phosphorylated as early as 15 min after Torin 1 treatment and appeared to reach a steady state shortly thereafter (Fig. 2c, d). The same effect was seen total medium starvation with Hank’s Balanced Salt Solution (HBSS), albeit at a slightly slower rate (Additional file 1b, c).

ULK1-mediated Beclin 1 phosphorylation is promoted by the presence of ATG14 [23]. To assess the requirement of Beclin 1 in ATG14 phosphorylation, we treated Beclin 1 KO MEF cells with Torin 1 and then examined ATG14 pS29 levels. The result showed that the ability of ULK1 to phosphorylate ATG14 S29 is severely impaired in Beclin 1 KO MEF cells, but that ATG14 pS29 levels recover when an exogenous Beclin 1 is reintroduced (Fig. 2e, f).

To test the specific role for ULK1/2 kinases in phosphorylating ATG14, we treated ULK1/2 double knockout (DKO) MEF cells with Torin 1. Torin 1-induced ATG14 phosphorylation was detected in wildtype MEF cells but not in ULK1/2 DKO MEF cells (Fig. 2g). Knocking out ATG14 completely abolished the phosphorylation signal after Torin 1 and HBSS incubation, indicating the specificity of the anti-pS29 ATG14 antibody (Fig. 2h). Thus our results demonstrate that ULK1-mediated phosphorylation of ATG14 S29 is regulated by the mTOR pathway.

### Phosphorylation of ATG14 S29 critically regulates Vps34 lipid kinase activity

**Fig. 3:**
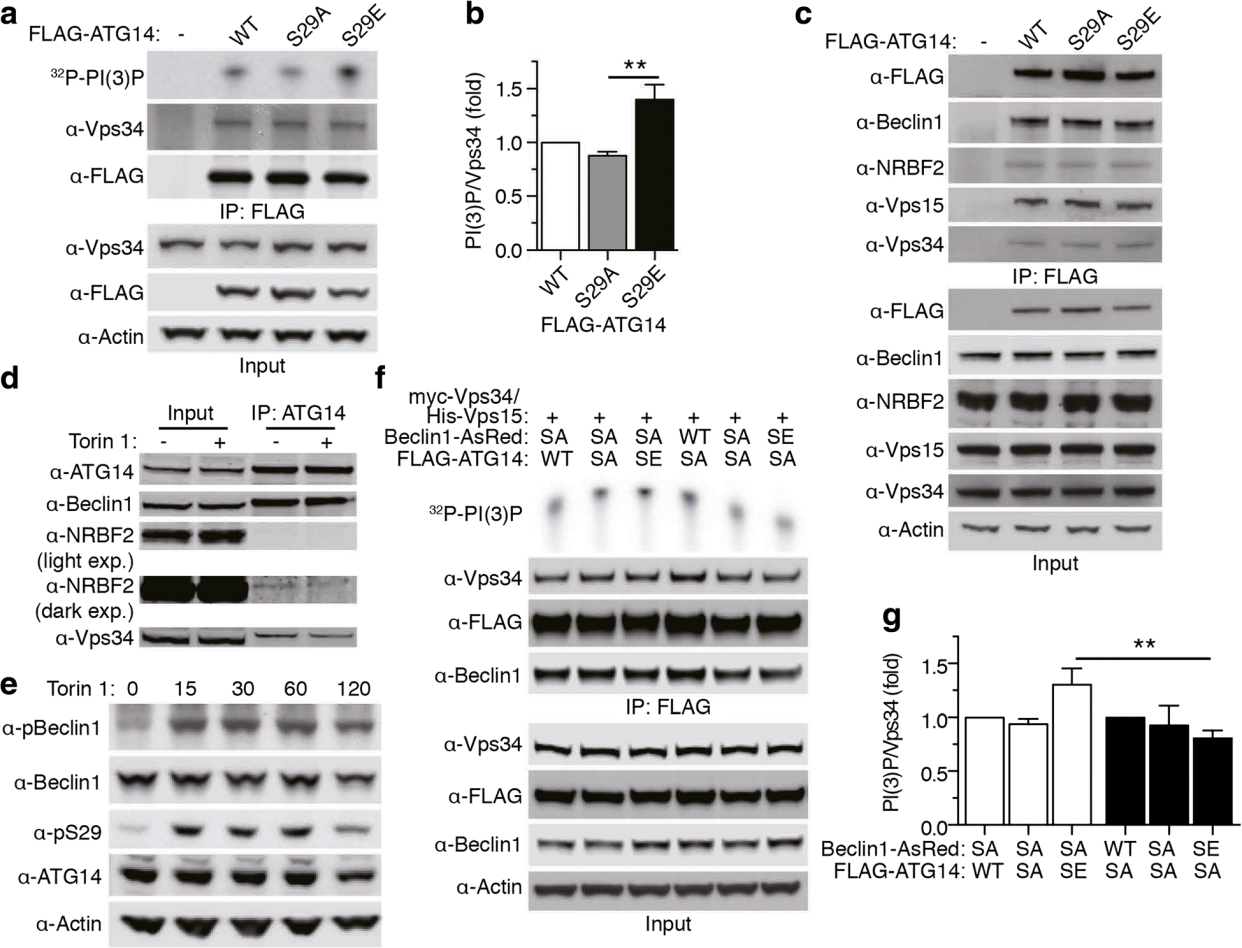
ATG14 phosphorylation promotes Vps34 lipid kinase activity. **a** Vps34 lipid kinase assay. Empty vector or FLAG-ATG14 S29 WT, A, or E mutants were overexpressed in HEK cells and pulled down using antibodies against FLAG. **b** Quantification of PI(3)P levels were normalized to levels of Vps34 in the IP. Values were then normalized to the WT condition. **p < 0.01 (*n* = 5). Data are represented as mean +/− SEM. **c** Co-immunoprecipitation of ATG14 and Vps34 complex members, Beclin 1, Vps34, Vps15 and NRBF2. Empty vector or FLAG-ATG14 S29 WT, A, or E mutants were overexpressed in HEK cells and pulled down using antibodies against FLAG. **d** Torin 1 treatment does not alter binding of Vps34 complex members. MEF cells were treated with Torin 1 for 60 min. Cell lysates were subject to IP using antibody against ATG14. **e** Phosphorylation levels of Beclin 1 and ATG14 from HCT116 cells treated with Torin 1 for 15, 30, 60, and 120 min. **f** Vps34 lipid kinase assay for Beclin 1 and ATG14 phosphorylation mutation comparison. Beclin 1-AsRed, FLAG-ATG14, and dual myc-Vps34 his-Vps15 plasmid were overexpressed and purified from HEK cells using antibodies against FLAG. **g** Quantification of PI(3)P levels were normalized to levels of Vps34 in the IP. Values were then normalized to the WT condition. Two-way ANOVA analysis with Bonferroni posttest was used for the effect of the nature of the mutation and which protein was being mutated. Main effect of Beclin 1 or ATG14 mutation F(1, 18) = 4.08, *p* = 0.0587; Main effect of type of mutation F(1, 18) = 0.71, *p* = 0.5043; Interaction F(2, 18) = 3.80, *p* = 0.0421. **p < 0.01 (*n* = 5). Data are represented as mean +/− SEM

To examine the role for pS29 of ATG14 in Vps34 kinase activity, we generated ATG14 phosphorylation mutants either to mimic phosphorylation (S/E) or suppress phosphorylation (S/A). FLAG-ATG14 S29E-associated Vps34 showed a significant increase in lipid kinase activity compared to that of FLAG-ATG14 S29A (Fig. 3a, b). Previous studies identified multiple protein components in Vps34 autophagy complex [11, 24, 25]. To test whether ATG14 pS29 interferes with the interaction of ATG14 and the other components, thus affecting Vps34 activity, we performed co-immunoprecipitation experiments from cells transfected with FLAG-tagged ATG14 variants. In examining the endogenous ATG14 binding proteins through FLAG pull-down, we detected no changes in the levels of Beclin 1, NRBF2, Vps15, or Vps34, comparing wildtype ATG14 to S29E or S29A mutants (Fig. 3c). Furthermore, treatment with Torin 1 had no effect on Beclin 1, Vps34, or NRBF2 binding to ATG14 (Fig. 3d). Thus the increased Vps34 activity caused by ATG14 pS29 is unlikely due to the change of ATG14 interactions with above components in the Vps34 complex.

Recent structural analysis of the Vps34 complex reveals that the N-termini of ATG14 and Beclin 1 are in close proximity [26, 27]. Beclin 1 is necessary for ATG14 phosphorylation and stabilization (Fig. 2e, [9]) and ATG14 is necassary for Beclin 1 phosphorylation [23]. Given the tight association of ATG14 and Beclin 1 proteins, we investigated the relationship between ATG14 and Beclin 1 phosphorylation. We found that ATG14 and Beclin 1 are phosphorylated following the same trend upon Torin 1 treatment (Fig. 3e). To distinguish the individual contributions of Beclin 1 and ATG14 phosphorylation to Vps34 activity regulation, we examined Vps34 activity in the presence of various combinations of ATG14 and Beclin 1 phosphorylation variants. FLAG-ATG14 WT, S29A, and S29E were co-expressed with Beclin 1-AsRed S14A and Vps34 was pulled down using antibodies against FLAG. Similarly, Beclin 1-AsRed WT, S14A, and S14E were co-expressed with FLAG-ATG14 S29A. Interestingly, ATG14 S29E is able to increase Vps34 activity despite the presence of Beclin 1 S14A. In contrast, Beclin 1 S14E is unable to enhance Vps34 activity in the presence of ATG14 S29A (Fig. 3f, g). Thus our result suggests that ULK1-mediated ATG14 phosphorylation is critical to promoting Vps34 activity.

### Mutant Huntingtin causes a reduction in ATG14 phosphorylation and ATG14-Vps34 kinase activity in HD mouse brain

**Fig. 4:**
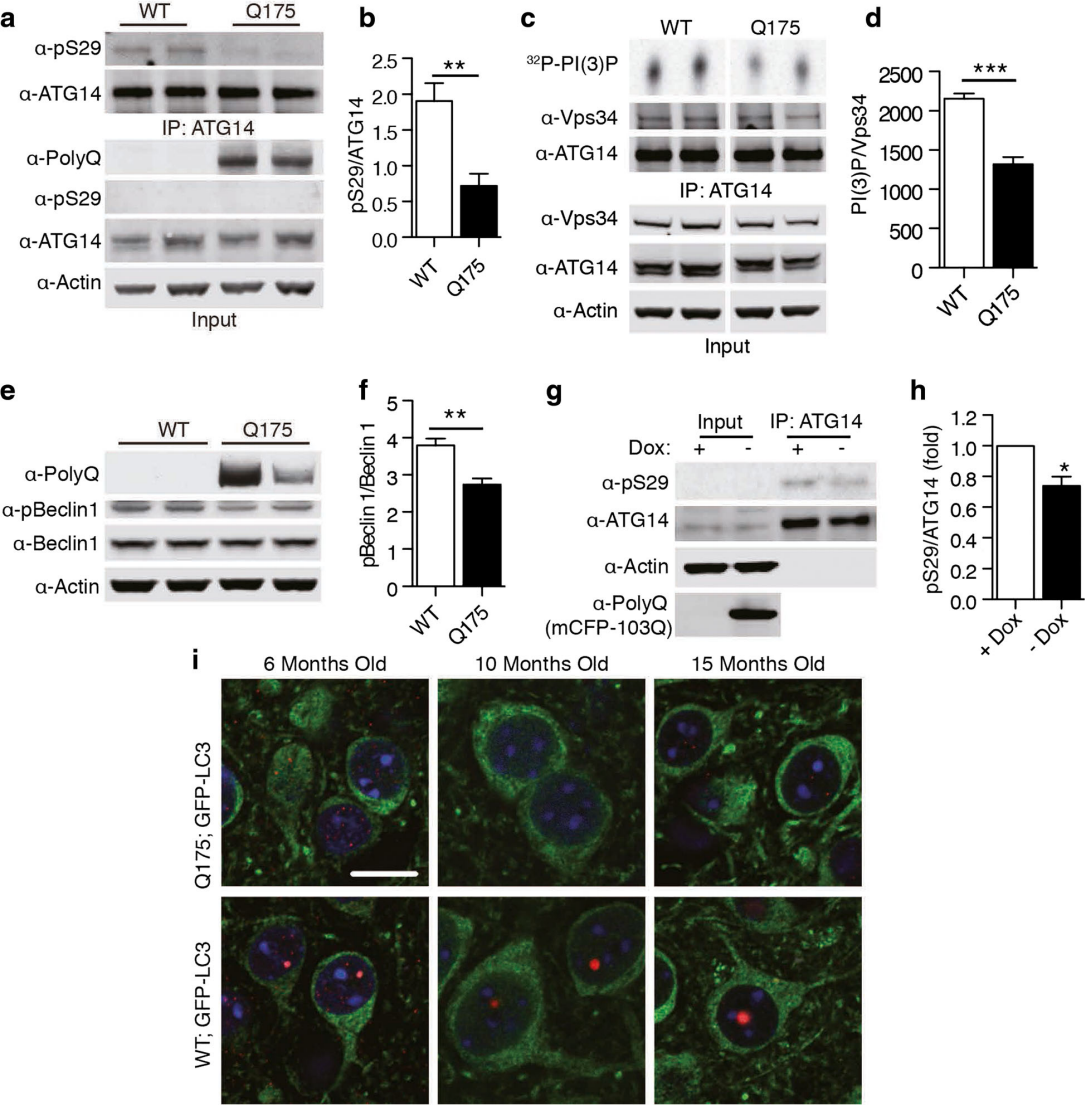
ATG14-associated Vps34 activity is reduced in Q175 brains. **a** ATG14 phosphorylation in Q175 brains. ATG14 was immunopurified using antibodies against ATG14. Two representative lanes shown for each experiment. **b** Quantification of ATG14 phosphorylation. **p < 0.01 (*n* = 5). Data are represented as mean +/− SEM. **c** In-vitro lipid kinase assay of Vps34 complex pulled down with antibodies against ATG14 from the brains of Q175 mice. Thin layer chromatography was used to separate ^32^P-PI(3)P. **d** Quantification of PI(3)P levels were normalized to levels of Vps34 in the IP. ***p < 0.001 (*n* = 4). Data are represented as mean +/− SEM. **e** Beclin 1 S14 phosphorylation in Q175 mice. **f** Quantification of Beclin 1 phosphorylation. **p < 0.01 (*n* = 5). Data are represented as mean +/− SEM. **g** ATG14 phosphorylation in mCFP-103Q expressing HeLa cells. Doxycycline was removed for 7 days to induce protein expression. IP was performed against ATG14. **h** Quantification of ATG14 phosphorylation. Values were normalized to control. *p < 0.05 (*n* = 4). Data are represented as mean +/− SEM. **I** Immunofluorecent imaging of autophagosome reporter GFP-LC3 distribution in striatal nuclei of Q175;GFP-LC3 and control WT;GFP-LC3 mice. Mice at 6, 10 and 15 months are examined. mHtt nuclear aggregate staining in red and GFP-LC3 in green. Scale bar 10 μm

In an earlier study, we identified an ULK1-mediated phosphorylation site on p62 [14]. During proteotoxic stress induced by expression of mutant Htt, p62 phosphorylation is increased in cell and mouse models of HD. We therefore sought to further dissect the autophagy pathway in HD using ATG14 phosphorylation as an autophagy measure. To do this, we used the Q175 mice, an HD model carrying a knock-in allele of mutant Huntingtin containing ~175 glutamine repeats [28]. Opposite to the reported increased p62 phosphorylation levels, we found that Q175 mice displayed a ~40% decrease in pS29 levels in the cortex, despite unchanged total ATG14 protein levels (Fig. 4a, b). We then assessed Vps34 activity, since ATG14 phosphorylation is sufficient to regulate its lipid kinase activity (Fig. 3). We pulled down ATG14-associated Vps34 complex from the cortex of Q175 mice using antibodies against ATG14. As expected, we found that ATG14-associated Vps34 activity was decreased in Q175 brain (Fig. 4c, d). We also examined the Beclin 1 phosphorylation, an ULK1 kinase substrate that was implicated in regulating Vps34 activity [23]. Western blot analysis showed a reduced level of Beclin 1 phoshp-Ser14 in Q175 brains compared to control mice (Fig. 4e, f). Thus our results suggest a decrease in ULK1-mediated regulation of ATG14-associated Vps34 kinase activity in Q175 brains.

We also examined ATG14 phosphorylation an HD cell model, which expresses the N-terminus of Htt following 103 polyQ repeats [13]. Cells expressing mCFP-103Q fragments also showed a decrease in ATG14 phosphorylation (Fig. 4g, h).

To understand autophagic activity in HD, we examined autophagosome formation in the Q175 mice, which expressed green fluorescence protein fused with LC3 (GFP-LC3) as reporter [18] after crossing GFP-LC3 mice to Q175 mice. Despite the expression of mutant polyQ-Htt protein, which accumulates into nuclear aggregates, there was little change in GFP-LC3 fluorescence intensity or distribution in 6, 10 or 15 month old mice, indicating no obvious change in autophagosome formation or block of autophagosome clearance (Fig. 4i). The observation is in contrast to the Lurcher mice, where mutant Purkinje cells produce a large number of GFP-LC3 associated autophagosomes prior to degeneration in response to excitotoxic stimuli [29]. They also differ from HdhQ200 mice (Knock-in), which are related to Q175 mice and display accumulation of perinuclear mHtt aggregates accompanied by the increase in autophagsome markers LC3 II and p62 protein levels [3]. Analysis of LC3II in Q175 brain, however, shows no obvious change of its protein levels compared to wildtype brain (Additional file 2 a, b). Together with the previous data indicating little change in p62 total levels in Q175 brain [14], the results therefore suggest no significant impairment of autophagic activity in the brain of Q175 mice. Thus the ~35% decrease of ATG14-linked Vps34 activity (Fig. 4c, d) does not corroborate a significance reduction of basal autophagy in the Q175 brain.

### Proteotoxic stress causes a reduction in ATG14 phosphorylation and redistribution of ULK1 to an insoluble cellular fraction

**Fig. 5:**
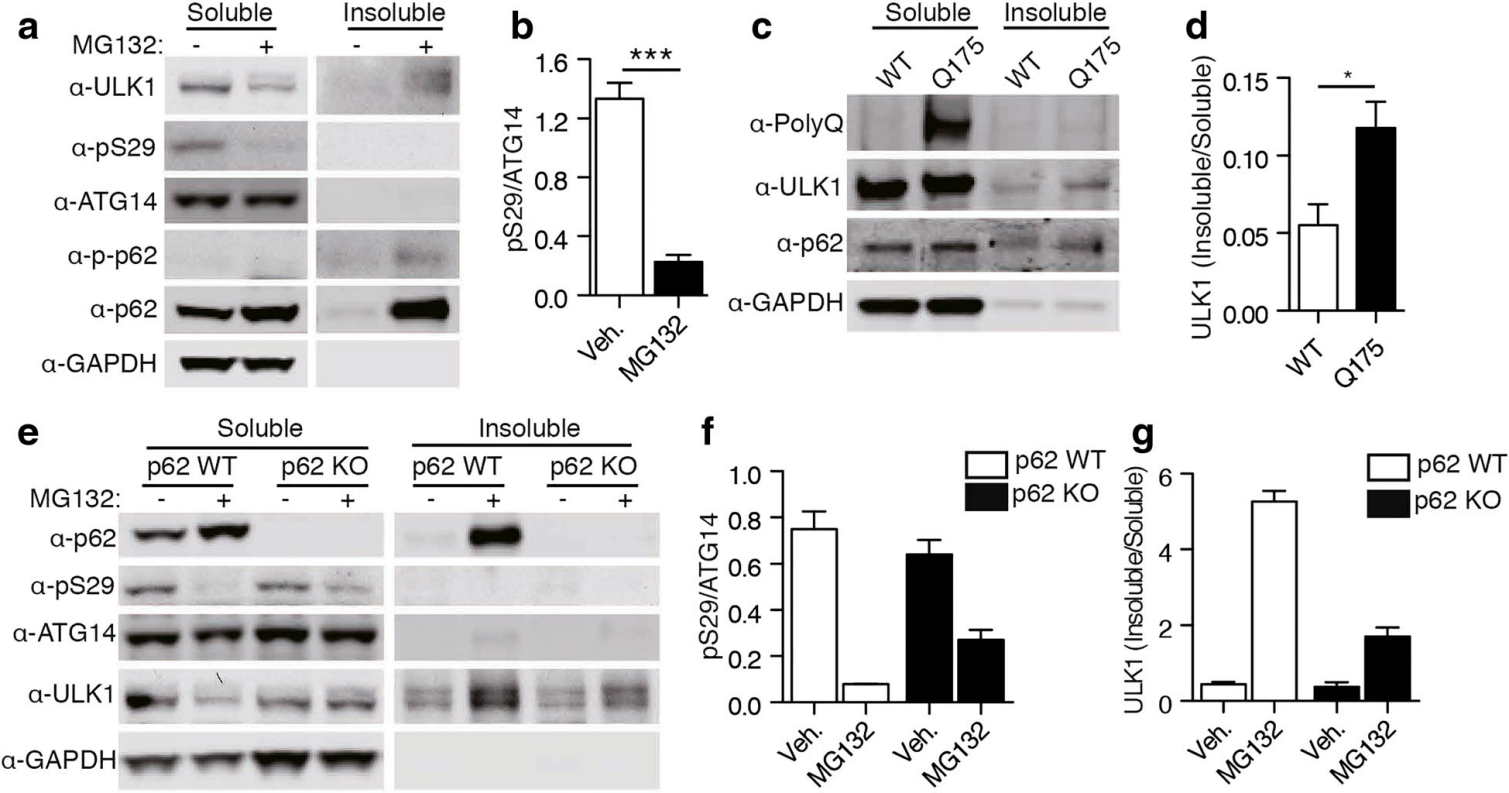
Proteotoxic stress causes a decrease in ATG14 phosphorylation and a redistribution of ULK1 into insoluble fraction. **a** ATG14 phosphorylation after proteasome inhibition. Phosphorylation of p62 is at S409. MEF cells were treated with MG132 for 16 h then separated into Triton X-100 soluble and insoluble fractions. **b** Quantification of ATG14 phosphorylation. ***p < 0.001 (*n* = 3). Data are represented as mean +/− SEM. **c** ULK1 accumulates in the Triton X-100 insoluble fraction in the brains of Q175 mice. **d** Quantification of ULK1 in the insoluble fraction. *p < 0.05 (*n* = 3). Data are represented as mean +/− SEM. **e** ATG14 phosphorylation after proteasome inhibition. P62 WT or KO MEF cells were treated with MG132 for 16 h then separated into Triton X-100 soluble and insoluble fractions. **f** Quantification of ATG14 phosphorylation in the soluble fraction. Two-way ANOVA was performed. Main effect of MG132 F(1, 8) = 92.60, *p* < 0.0001; Main effect of p62 KO F(1, 8) = 0.56, *p* = 0.4748; Interaction F(1, 8) = 7.82, *p* = 0.0233. (*n* = 3). Data are represented as mean +/− SEM. **g** Quantification of ULK1 in the insoluble fraction. Main effect of MG132 F(1, 8) = 252.94, *p* < 0.0001; Main effect of p62 KO F(1, 8) = 87.94, *p* < 0.0001; Interaction F(1, 8) = 81.33, *p* < 0.0001 (*n* = 3). Data are represented as mean +/− SEM

Proteotoxic stress induced by proteasomal inhibition also promotes ULK1-mediated phosphorylation of p62/SQSTM1, an autophagy receptor in selective autophagy [14]. To understand the impact of proteotoxic stress on ATG14 phosphorylation, we treated MEF cells with MG132, a proteosomal inhibitor. Similar to the effect of mutant Htt expression, MG132 caused a marked decrease in ATG14 p-S29 (Fig. 5a, b). As expected, p62 accumulated in the insoluble fraction, but ATG14 did not. Consistent with our previous results, there was also a decrease in soluble ULK1 levels but an increase in insoluble ULK1 levels (Fig. 5a; [14]). In Q175 mice, we also see a similar accumulation of ULK1 in the insoluble fraction (Fig. 5c, d), albeit to a lesser degree. This could be a result of the difference between cell type homogeneity and mutant Htt expression in the Q175 brain.

Given that ULK1 phosphorylates both p62 and ATG14, it is surprising that these signaling events appear to be differentially regulated. Moreover, we showed previously that p62-ULK1 binding is increased in the presence of mutant Htt or proteotoxic stress [14]. We therefore hypothesize that the differential regulation can be, in part, explained by p62 competing with ATG14 (both as substrates) for ULK1 kinase activity. To test this hypothesis, we examined pS29 ATG14 levels in wildtype and p62 KO MEF cells after MG132 treatment. We observed higher levels of ATG14 phosphorylation in p62-depleted cells treated with MG132 when compared to WT (Fig. 5e, f). Interestingly, we also notice less ULK1 in the insoluble fraction in p62 KO MEF compared to control cells (Fig. 5g), consistent with the loss of p62 competition or sequestration, which would otherwise recruit ULK1 into the insoluble fraction.

ATG13 and FIP200 are components of ULK1 complex and important for ULK1 kinase regulation. We then tested whether MG132 can alter the binding of ATG13 and FIP200 with ULK1 and localization of ATG13 and FIP200, which may account for the decrease of ATG14 phosphorylation. We found that MG132 treatment does not alter the interaction between ULK1 and ATG13 or FIP200 as assayed by co-IP; however, MG132 treatment causes FIP200 and ATG13 redistribution into insoluble fraction (Additional file 3 a, b). Thus the result suggests that p62 sequestration of ULK1-ATG13-FIP200 complex into insoluble fraction leads to reduced ATG14 phosphorylation.

### ULK1-mediated ATG14 phosphorylation promotes disease-related polyQ protein degradation

**Fig. 6:**
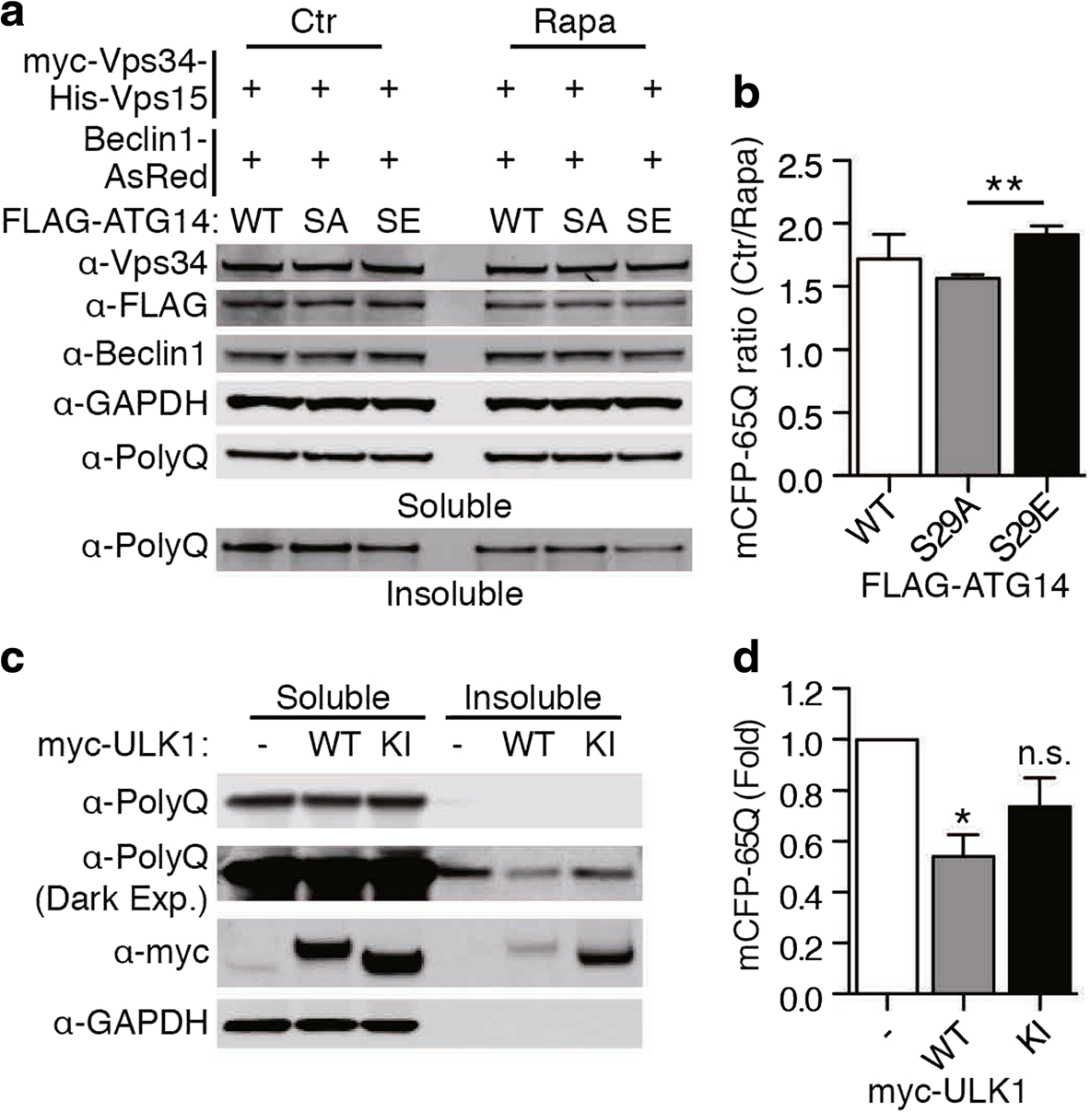
ULK1 activity regulates the degradation of polyQ proteins. **a** FLAG-ATG14 S29 WT, SA, or SE, Beclin 1-AsRed, and dual myc-Vps34 his-Vps15 plasmid were overexpressed in mCFP-65Q expressing HeLa cells then treated with rapamycin overnight. Cell lysates were separated into soluble and insoluble fractions. PolyQ was measured in the insoluble fraction. **b** Quantification of polyQ degradation in the insoluble fraction. Degradation was calculated as the ratio of control sample vs. rapamycin sample **p < 0.01 (*n* = 4). Data are represented as mean +/− SEM. **c** Myc empty vector, myc-ULK1 WT or KI were overexpressed in mCFP-65Q expressing HeLa cells and cells were lysed 48 h after transfection. Cell lysates were separated into soluble and insoluble fractions. PolyQ was measured in the insoluble fraction. **d** Quantification of polyQ levels in the insoluble fraction. Values were normalized to empty vector condition. *p < 0.05 n.s. not significant (*n* = 4). Data are represented as mean +/− SEM

Previous evidence indicated a role for Vps34 activity in HD: blocking Vps34 activity increases the levels of polyQ protein [30, 31], while addition of synthetic PI(3)P, the Vps34 product, aids in the clearance of polyQ protein [13]. Given the role of autophagy in the clearance of protein aggregates, we next asked if pS29 ATG14 facilitates the removal of polyQ proteins. We overexpressed the ATG14 variants along with other Vps34 complex components in mCFP-65Q expressing HeLa cells as we described in our earlier study [14]. The result shows increased degradation of insoluble mCFP-65Q in the presence of S29E ATG14, compared to S29A, supporting the idea that ATG14 pS29 promotes the degradation of polyQ mutant (Fig. 6a, b).

Because our data showed that reducing p62 levels partially restores ATG14 phosphorylation, we hypothesized that ULK1 is a limiting factor in autophagic clearance of polyQ protein. We therefore asked if an increase in overall ULK1 kinase level could provide a beneficial role for the autophagic clearance of mutant Htt. To this end, we overexpressed the WT or KI mutant of ULK1 in mCFP-65Q cells. We found that overexpression of ULK1 WT but not ULK1 KI was able to effectively reduce insoluble mCFP-65Q protein levels (Fig. 6c, d). Taken together, our result provides evidence for ULK1-mediated ATG14 phosphorylation as a potential drug target for HD by enhancing autophagic clearance of mutant Htt aggregates.

## Discussion

Autophagy modulation is being pursued as a therapeutic strategy for neurodegenerative diseases associated with protein aggregates. Thus, a better understanding of the autophagy status in the disease process is critical to this endeavor. Our study provides an insight into the mechanism of autophagy regulation whereby ULK1/2 signals to autophagy-specific Vps34 complex to activate autophagy through the mTOR nutrient pathway. We find that ULK1 mediates ATG14 phosphorylation at a novel serine site, S29, in response to amino acid (but not glucose) starvation, which in turn up-regulates Vps34 kinase activity. Importantly, we find that the specific ATG14 phosphorylation and ATG14-associated VSP34 activity, which is autophagy-specific, are impaired in HD cell and animal models. Interestingly, our current assays suggest no obvious impairment of autophagic activity in the brain of Q175 mice. Thus the 35% decrease of ATG14-linked Vps34 activity does not corroborate a significance reduction of basal autophagy in the HD animal models. Note that our autophagy assays are based on GFP-LC3 reporter and protein levels of several autophagy markers including p62 [14], LC3II, ULK1, and Beclin 1-VPS34-ATG14 protein levels; they are limited in that they do not reflect accurately the autophagy flux, which has been extremely challenging to study in vivo. It is possible that transient, subtle and regional changes of autophagy in Q175 mice are difficult to detect using available approaches with low sensitivity. We thus postulate that monitoring ATG14 phosphorylation and ATG14-Vps34 activity provides a new means for in-vivo autophagy pathway analysis which serves as a useful biomarker for altered autophagy signaling in disease.

Interestingly, a previous study in HdhQ200 knockin model, which manifests an accelerated and robust phenotypes than Q175 mice, has perinulear mHtt foci accompanied by colocalized LC3 and p62 aggregates. The HdhQ200 mice have increased levels of LC3II and p62 levels in the striatal neurons [3]. Although interpreted as the induction of autophagy, the available evidence indeed informs little regarding the exact direction change of autophagy or autophagy flux, nonetheless shows the alteration of autophagy in HdhQ200 mice. Our study found no perinuclear mHtt in Q175 HD brain, which may explain the lack of GPF-LC3 puncta formation. The lack of GFP-LC3 puncta in control *GFP-LC3* mice is consistent with the idea that basal autophagosome formation is extremely rare in wildtype neurons as described by Mizushima’s group [3]. Then it is not surprising that no GFP-LC3 puncta formation was found in Q175 neurons, as the reduced ATG14 phosphorylation and Vps34 activity as shown in our study predicts even lower rate for autophagosome formation. It is likely that accelerated accumulation of mHtt aggregates in HdhQ200 mice causes apparent change in LC3 and p62 levels. It would be interesting to investigate the ULK1-mediated ATG14 and Beclin 1 phosphorylation in HdhQ200 mice. Furthermore, future study should examine whether a long term impact (e.g. >15 months) of the decrease of ATG14-linked Vps34 activity can result in detectable reduction of autophagy measured through autophagic markers such as LC3 and p62.

ULK1-mediated phosphorylation of ATG14 may represent an important signaling event for autophagy induction that coordinates the action of the two key components of autophagy machinery, the ULK1 kinase complex and ATG14-Vps34 complex (autophagy specific). During the preparation of this manuscript, Park, et al. published their independent discovery of ATG14 phosphorylation by ULK1 [32]. Their results are in an agreement with our conclusion that Vps34 activity is regulated by ATG14 phosphorylation and demonstrate its positive effects on autophagosome formation. A previous study showed that ULK1-mediated Beclin 1 phosphorylation contributes to the regulation of Vps34 activity [23]. However, it showed that ULK1-mediated Beclin 1 phosphorylation is also promoted by both UVRAG and ATG14. However, two separate reports reveal that starvation-induced mTOR inhibition either has no effect on UVRAG-Vps34 activity or decreases it by 60% [33, 34], raising a question about the precise role for phosphorylation of Beclin 1 in upregulating Vps34 activity. Our current data shows that, although phosphorylation of ATG14 and Beclin 1 occur with the similar kinetics in response to mTOR inhibition, ATG14 phosphorylation status is able to regulate Vps34 activity independent of Beclin 1 phosphorylation. Although it goes beyond the scope of the present study, further clarification of the role of Beclin 1 phosphorylation for UVRAG-Vps34 activity is warranted. Finally, given ATG14’s role at the ER-mitochondria contact site (MAM) and the requirement of ULK1 in ATG14 puncta formation during autophagy induction, it is possible that ULK1 activity is involved in the recruitment of one or more Vps34 complex members to the MAM [12, 35].

Our detailed study of ULK1 kinase and ATG14-Vps34 lipid kinase show reduced activity of both kinases, suggesting altered regulation of the autophagy pathway in HD models. Both phosphorylated Beclin 1 and ATG14 levels are decreased in HD brains, indicating the lower ULK1 kinase activity, which is regulated by mTOR nutrient pathway. The reduced ULK1-mediated ATG14 or Beclin 1 phosphorylation was initially surprising, given that ULK1-mediated p62 phosphorylation (mTOR-independent) increases in the same models of HD [14]. Interestingly, this differential ULK1 activity (mTOR vs. non-mTOR regulated) was also seen in cultured cells following proteasomal stress. It is possible that the regulatory mechanism for the aberrant ULK1 activity is shared between proteasomal stress and HD. Our data suggests that autophagy receptor p62 plays a role in regulating ULK1-mediated ATG14 phosphorylation upon proteotoxic stress. From our earlier study, in both HD (expressing polyQ-protein) and proteasomal stress conditions, there is an increased binding between p62 and ULK1, concurrent with competition of ULK1 by p62 oligomerization [14]. As a potential result, the access of ATG14-Vps34 complex to ULK1 kinase may be reduced. Our result also indicates proteasomal stress condition causes sequestration of the whole ULK1-ATG13-FIP200 complex. A similar competition model for ULK1 binding was also proposed by another group, in which Htt was shown to pull ULK1 away from the inhibitory effects of mTOR during selective autophagy [36]. Alternatively, optineurin, another autophagy receptor, is involved in aggrephagy and has also been shown to interact with Htt and co-localize with ULK1 [37–40]. Therefore, we are unable to rule out the possibility that other autophagy receptors may also sequester ULK1 away from ATG14 during proteotoxic stress.

Finally, our current work extends our previous report that ULK1-mediated p62 phosphorylation in selective autophagy helps clear polyQ protein [14] by showing that ATG14 phosphorylation and ULK1 activity per se has beneficial effects in the clearance of mutant Htt. By targeting ULK1 activity to regulate autophagy, the unintended consequences of general mTOR inhibition can be avoided. Future work should be aimed at exploring the modulation of ULK1 activity for the treatment of neurodegenerative diseases associated with protein aggregates.

## Conclusions

As the major pathway for protein aggregate clearance, autophagy has been heavily implicated in the HD pathogenesis and considered a therapeutic target. However, the exact molecular mechanisms at work remain elusive. In this work, we identify a critical process during autophagosome biogenesis that is disregulated following mutant Htt-induced proteotoxic stress. We find that in the canonical autophagy pathway, ULK1 phosphorylates ATG14, which promotes Vps34 lipid kinase activity. ATG14 phosphorylation and Vps34 activity, however, are impaired in HD models. Our study highlights ULK1 and Vps34 as potential targets for the treatment of HD.

## Additional files

Additional file 1: ATG14 serine 29 **a** HCT116 cells were cultured in glucose free medium for 12 h. **b** ATG14 phosphorylation after 30, 60, 120, and 240 min of HBSS treatment in HCT116 cells. **c** ATG14 phosphorylation levels were normalized to total ATG14 levels and compared to the control condition. A one-way ANOVA with Bonferroni’s posttest was performed. F(4, 10) = 11.68, *p* = 0.0009. ****p* < 0.001 ***p* < 0.01. Data are represented as mean +/− SEM. (PDF 501 kb)
Additional file 2: LC3-II levels are unchanged in Q175 striatal region (15 months) **a** Western blot of LC3 and PolyQ from the striatum of Q175 mice. **b** Quantification of LC3-II normalized to actin. n.s. not significant (*n* = 4). Data are represented as mean +/− SEM. (PDF 431 kb)
Additional file 3: Interaction and redistribution of ULK1, ATG13 and FIP200 following the treatment of MG132. **a**. ULK1/2 WT and DKO MEFs treated with MG132 were investigated for interactions between ULK1, ATG13 and FIP200 by IP with ULK1 antibody. **b** Westernblot analysis of ULK1, ATG13 and FIP200 protein levels in cell fractions collected from MG132-treated cells. No change in ULK1, ATG13 and FIP200 interaction as well as their redistribution in soluble vs. insoluble fractions was observed. (PDF 503 kb)

## Abbreviations

HD: Huntington’s disease
PI(3)P: phosphatidylinositol 3-phosphate
PI3KC3: Class III phosphoinositide 3-kinasepolyQ, poly-glutamine
ULK1: Unc-51 Like Autophagy Activating Kinase 1
