# Supplementary figures and images for: ULK1-mediated phosphorylation of ATG14 promotes autophagy and is impaired in Huntington’s disease models
Source: Mol Neurodegener. 2016 Dec 9;11:76. doi: 10.1186/s13024-016-0141-0 (PMC5148922; doi:10.1186/s13024-016-0141-0)

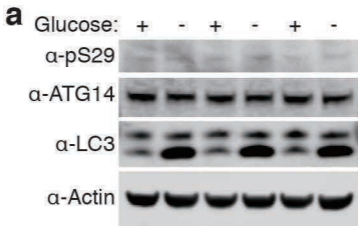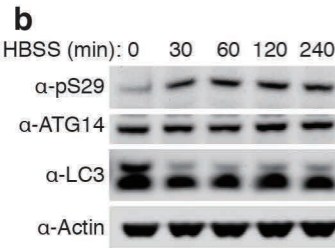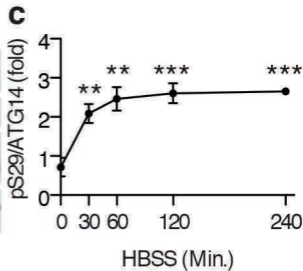

Supplement: Additional file 1: — ATG14 serine 29 a HCT116 cells were cultured in glucose free medium for 12 h. b ATG14 phosphorylation after 30, 60, 120, and 240 min of HBSS treatment in HCT116 cells. c ATG14 phosphorylation levels were normalized to total ATG14 levels and compared to the control condition. A one-way ANOVA with Bonferroni’s posttest was performed. F(4, 10) = 11.68, p = 0.0009. ***p < 0.001 **p < 0.01. Data are represented as mean +/− SEM. (PDF 501 kb) [file 13024_2016_141_MOESM1_ESM.pdf]

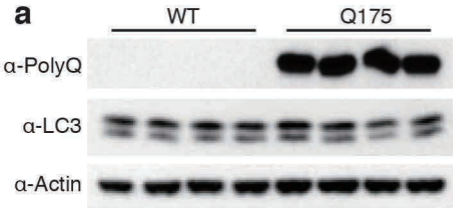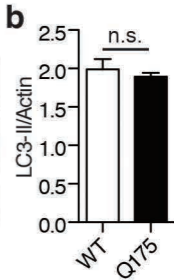

Supplement: Additional file 2: — LC3-II levels are unchanged in Q175 striatal region (15 months) a Western blot of LC3 and PolyQ from the striatum of Q175 mice. b Quantification of LC3-II normalized to actin. n.s. not significant (n = 4). Data are represented as mean +/− SEM. (PDF 431 kb) [file 13024_2016_141_MOESM2_ESM.pdf]

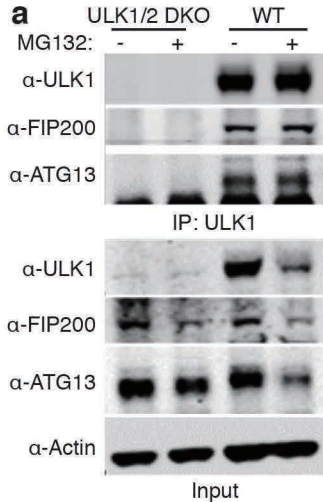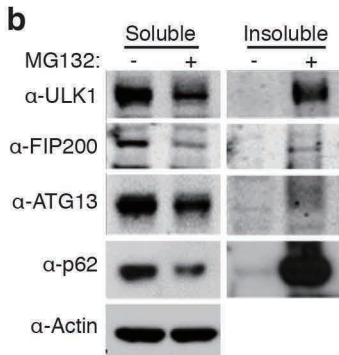

Supplement: Additional file 3: — Interaction and redistribution of ULK1, ATG13 and FIP200 following the treatment of MG132. a. ULK1/2 WT and DKO MEFs treated with MG132 were investigated for interactions between ULK1, ATG13 and FIP200 by IP with ULK1 antibody. b Westernblot analysis of ULK1, ATG13 and FIP200 protein levels in cell fractions collected from MG132-treated cells. No change in ULK1, ATG13 and FIP200 interaction as well as their redistribution in soluble vs. insoluble fractions was observed. (PDF 503 kb) [file 13024_2016_141_MOESM3_ESM.pdf]
